# Gastric cancer following a liver transplantation for glycogen storage disease type Ia (von Gierke disease): A case report

**DOI:** 10.3892/ol.2014.2599

**Published:** 2014-10-09

**Authors:** HUA XIAO, JIANMIN BIAN, LEI ZHANG, ZHAOMING WANG, AIXING DING

**Affiliations:** Department of General Surgery, Nanjing First Hospital, Nanjing Medical University, Nanjing, Jiangsu 210006, P.R. China

**Keywords:** glycogen storage disease, *de novo* gastric cancer, liver transplantation

## Abstract

Glycogen storage disease type Ia (GSD-Ia; also termed von Gierke disease) is an inherited metabolic disorder resulting from a glucose-6-phosphatase deficiency. Liver transplantation is considered to be the most effective treatment for GSD-Ia patients. In the present study, the case of a patient with GSD-Ia who received a liver transplantation at 17 years of age is presented. During the 12 years following transplantation, the patient’s quality of life markedly improved. However, recently, the patient was diagnosed with *de novo* gastric cancer following a biopsy. Thus, a total gastrectomy with lymph node dissection was performed and the tumor was histologically determined to be a poorly differentiated adenocarcinoma (histopathological stage, pT4N1M0). The patient recovered well and was discharged on postoperative day 10 without any complications. To the best of our knowledge, this is the first case of *de novo* gastric cancer in a patient with GSD-Ia to be reported.

## Introduction

Glycogen storage disease type Ia (GSD-Ia; also termed von Gierke disease) is an inherited metabolic disorder that is caused by a deficiency of glucose-6-phosphatase, which leads to the limited production of free glucose, and an excessive accumulation of glycogen in the liver, kidney and intestinal mucosa. The major short-term manifestations of the disease include hypoglycemia, lactic acidemia and hepatomegaly. By contrast, the development of hepatocellular adenoma presents a common long-term complication of the disease (1,2). As a result of these complications, liver transplantation is considered to be the most effective treatment for GSD-Ia patients (3). To the best of our knowledge, no cases of *de novo* gastric cancer in GSD-Ia patients following liver transplantation have been reported. In the current study, the rare case of a 29-year-old male who developed *de novo* gastric cancer, following an orthotopic liver transplantation (OLT) twelve years ago for the treatment of GSD-Ia is presented. Written informed consent was obatined from the patient’s family.

## Case report

**Figure 1:**
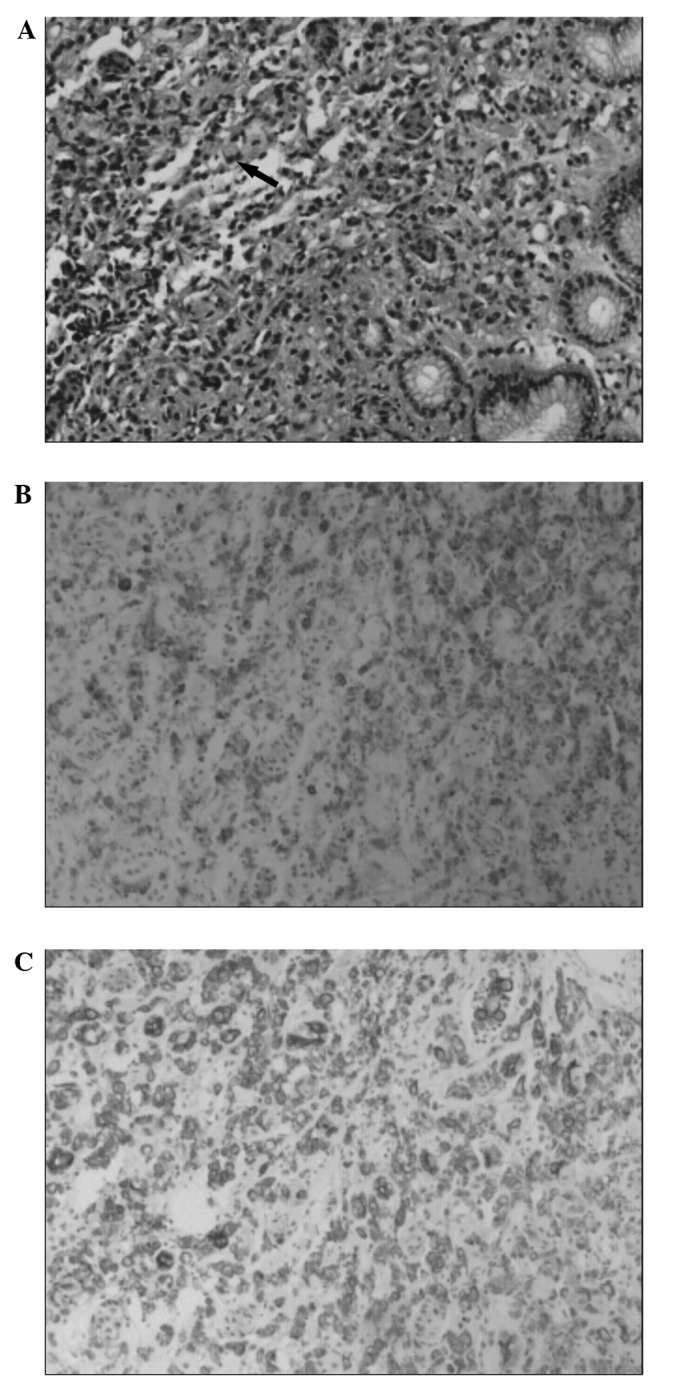
Observations of the biopsied specimen obtained via gastric endoscopy. (A) Histological examination found signet-ring cells in the muscularis propria (black arrow). Immunochemistry revealed the positive expression of (B) epithelial membrane antigen and (C) cytokeratin (stain, anti-cytokeratin antibody; magnification, ×100).

**Figure 2:**
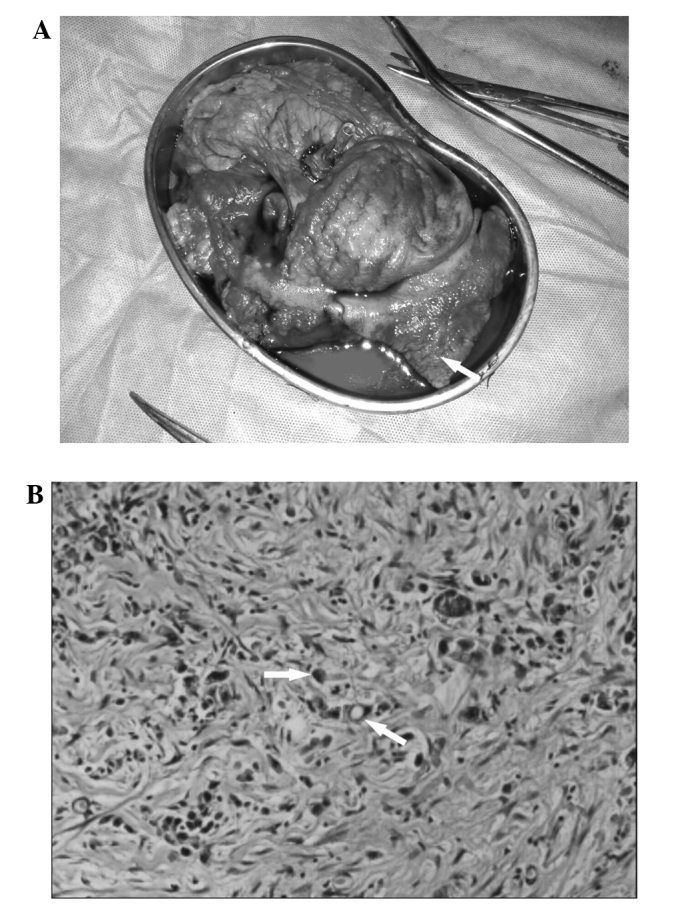
(A) Surgical findings revealed that the gastric posterior wall was extensively rigid and the margins were irregular (white arrow). (B) Histopathological examination of the resected tissue indicated poorly differentiated adenocarcinoma and a number of signet-ring cell carcinoma cells (white arrows) (stain, hematoxylin and eosin; magnification, ×100).

In June 2001, a 29-year-old male underwent a modified piggy-back liver transplantation for von Gierke disease. Following the surgery, the patient’s quality of life markedly improved. Tacrolimus (3 mg, every 12 h for one year), mycophenolate mofetil (1000 mg, every 12 h for six months), and prednisone (5 mg, every 12 h for two months) were administered for immunosuppression following surgery and currently, the patient receives 50 mg Ciclosporin in the morning and 75 mg at night, daily. In August 2013, the patient was admitted to Nanjing First Hospital (Nanjing, China) due to melena that had been occurring for three days. The melena disappeared following conservative treatment, which included hydration with water, glucose, electrolytes and amino acids, fasting and the administration of Losec, a proton-pump inhibitor. A gastric endoscopy was subsequently performed and revealed a deep ulcer with a necrotic gray area in the base, which was located in the posterior wall of the fundus region of the stomach, accompanied by edema in the immediately adjacent mucosa. Histological examination of the biopsied specimen revealed signet-ring cells in the muscularis propria (Fig. 1A) and immunochemical staining was positive for epithelial membrane antigen (Fig. 1B) and cytokeratin (Fig. 1C) expression, which indicated the diagnosis of signet-ring cell carcinoma. Enhanced computed tomography scanning and magnetic resonance imaging of the upper abdomen revealed a thickened stomach wall. In addition, pre-operative serological examination revealed that the α-fetoprotein (3.46 ng/ml; normal range, <20 ng/ml), carcinoembryonic antigen (2.37 ng/ml; normal range, <10 ng/ml) and carbohydrate antigen 19-9 (12.58 U/ml; normal range, <37 U/ml) levels were normal. Following the diagnosis of *de novo* gastric cancer, surgery was performed in September 2013. During the laparotomy, a broad region of the gastric posterior wall was identified to be rigid with irregular margins (Fig. 2A). The tumor measured ~9.5×8.5 cm in diameter. Subsequently, a total gastrectomy with lymph node dissection was performed. Reconstruction was performed using the Rous-en-Y esophagojejunostomy method and a naso-intestinal feeding tube was inserted into the distal bowel. Following surgery, routine nutritional support and immunosuppressive medications were administered. Histopathological examination of the resected stomach tissue revealed poorly differentiated adenocarcinoma with a number of partially signet-ring carcinoma cells (Fig. 2B); the invasion penetrated the wall via the serosa with perigastric lymph node metastasis (1/11 lymph nodes removed from the lesser curvature and 0/4 lymph nodes from the greater curvature were pathologically confirmed to be metastatic). The tumor was staged as pT4N1M0 with no distant metastasis. The patient recovered well and was discharged on postoperative day 10 without any complications.

## Discussion

*De novo* stomach cancer development following liver transplantation is relatively rare. However, the incidence of *de novo* malignancy following OLT is significantly higher in patients that have undergone OLT, than that in the general population. Park *et al* (4) retrospectively reviewed 1,952 patients following OLT and found that 44 patients exhibited *de novo* malignancies following a mean post-transplantation period of 41 months. Among them, 11 patients were diagnosed with gastric cancer. The relative risk of malignancy following OLT was 7.5-fold higher than that of the general population. However, the majority of patients with recurrent malignancies underwent OLT for hepatocellular carcinoma (HCC), rather than for benign liver diseases.

Independent risk factors for the development of a *de novo* malignancy include individual pre-transplant disease status, immunosuppressive therapy and the time that elapses following OLT. With regard to pre-transplant disease status, numerous patients are diagnosed with HCC prior to OLT (5). Previous studies have indicated that individuals with a primary carcinoma are more susceptible to the development of additional tumors than healthy individuals, which may be a result of a genetic predisposition to malignancy (6–8). Furthermore, OLT recipients must undergo lifelong immunosuppression following OLT. The immune system is important in tumor surveillance *in vivo* and suppression of immune system activity may promote tumorigenesis. Notably, immunosuppressive agents exhibit direct carcinogenic effects, which may induce cancer progression (9). Thus, it is biologically plausible that immunosuppression is associated with a higher incidence of malignancy when compared with the immunocompetent population. Finally, due to advances in the medical field, the survival time following transplantation has been prolonged significantly and thus, the risk of developing malignancy is increased. Previous studies have reported that the longest interval observed between transplantation and occurrence of *de novo* gastric cancer was 70 months (10–12). In the present case, the *de novo* malignancy occurred 12 years following transplantation and, therefore, is the longest interval reported thus far.

Previous studies have reported that smoking and alcohol consumption may also contribute to the development of *de novo* gastric cancer (9,12,13). Acute alcohol intoxication is hypothesized to decrease natural killer cell activity and promote tumor metastasis (12). In the present study, the patient’s irregular eating habits, including irregular eating patterns and consuming ‘unhealthy’ food, were considered to increase the risk of developing *de novo* malignancy as this may destroy the gastric muscousal membrane, leading to malignancy (14,15). Furthermore, primary stomach diseases, including chronic gastritis, stomach ulcers and the *Helicobacter pylori* infection may also be pathogenic factors. In the present case, the patient was diagnosed with chronic gastritis following a gastric endoscopy and was found to be positive for the *H. pylori* infection.

At present, no standard treatment regimen has been recommended for *de novo* gastric cancer in the literature or guidelines; thus, clinicians usually follow the treatment principles for gastric cancer. Numerous factors require particular attention with regard to the treatment of patients; further investigation is required regarding the immunosuppressive agents that are administered, particularly whether they should be sustained during the perioperative period. In the current case, the patient received continuous low-dose immunosuppressive medications and no postoperative complications, such as infection or disunion incisions were identified. The adoption of adjuvant chemotherapy remains controversial, as chemotherapy may harm the liver. In addition, the patient’s medical history, including the specific transplantation method, must be investigated prior to surgery for gastric cancer so that the appropriate approach is selected to reduce the negative impact of surgery. In the present case, a bilateral subcostal incision with midline extension toward the xiphoid was performed during the transplantation. Access to the abdominal cavity was via a median incision to avoid tissue adherence. During surgery, the anatomical structures of the hepatic hilum and hepatogastric region must be considered. Furthermore, when dissecting the lymph nodes, surgeons must consider the reconstructive vessels and bile ducts to avoid iatrogenic injury. In addition, liver function must be monitored following surgery. Enteral nutrition must be administered at an early stage using an intestinal feeding tube, while immunosuppressants may be administered via a nasogastric tube.

In conclusion, OLT recipients exhibit a higher risk of developing malignancies following a transplant. In this study, although GSD-Ia is a benign disease, with a good prognosis following effective treatment, the patient developed gastric cancer 12 years following OLT. D*e novo* malignancies usually develop quickly and are associated with a poor prognosis. Surveillance protocols are required for these patients in order to detect *de novo* tumors at an early stage. Subsequently, an appropriate surgical procedure must be performed so that the survival rate is improved.
